# Non-communicable eye diseases: facing the future

**Published:** 2014

**Authors:** Serge Resnikoff, Ivo Kocur

**Affiliations:** President, International Health and Development, Geneva, Switzerland. Serge.resnikoff@gmail.com; Medical Officer, Prevention of Blindness and Deafness, World Health Organization, Geneva, Switzerland. kocuri@who.int

**Figure:**
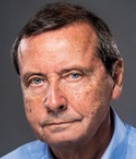
Serge Resnikoff

**Figure:**
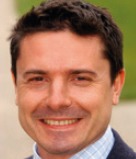
Ivo Kocur

The World Health Assembly, which met in May 2013, adopted a new global programme document entitled ‘Universal eye health: a global action plan 2014–2019’. The World Health Assembly is attended by ministerial delegations of all World Health Organization (WHO) Member States every year, and is where issues of major global public health importance are discussed. The global programme document was first drafted as part of an open, participatory process and was then discussed by the WHO Member States at the meeting. It states the major priorities for the global prevention of blindness efforts for the next five years.

**Figure:**
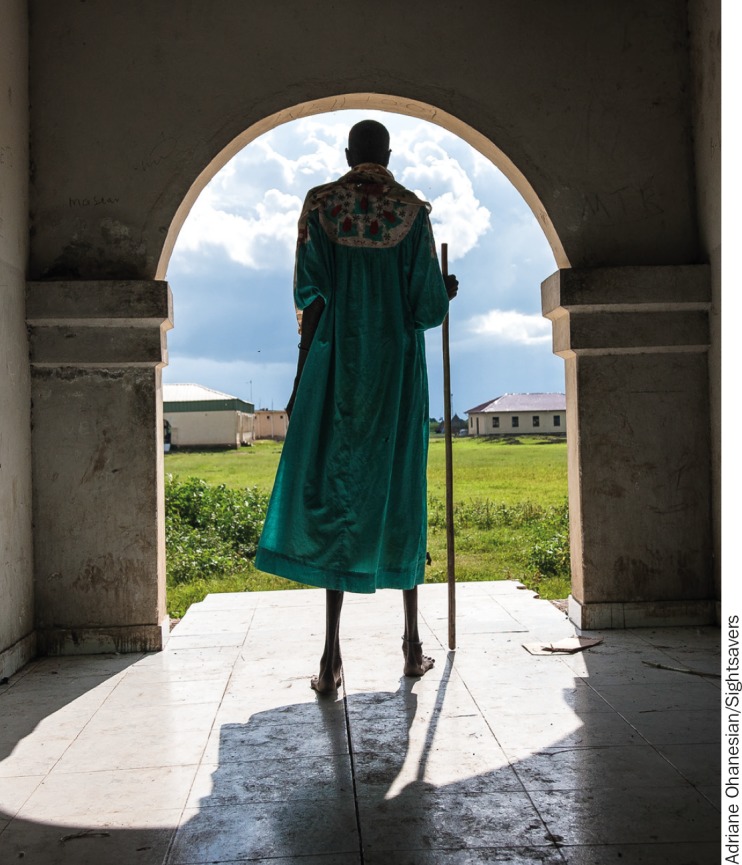
Diseases at the back of the eye are affecting older people in low-income countries

Cataract and uncorrected refractive errors remain by far the leading causes of visual impairment. However, several other eye conditions have emerged as significant threats to people's vision – those which are non-communicable (or chronic) and which become more prevalent with ageing. They are:

- diabetic retinopathy (DR)
- glaucoma
- age-related macular degeneration (AMD).

These diseases all affect the back of they eye. What they have in common is that they are incurable and require ongoing management (unlike cataract or refractive error, for which surgery or a pair of spectacles can restore vision).

Over the last two decades, these non-communicable eye diseases (NCEDs) have become much more significant.

1. As a result of health care initiatives, the proportion of people blind due to infectious eye diseases has decreased dramatically from 20% to 2% over the last three decades; the proportion due to other eye conditions (including NCEDs) has therefore increased.
2. People are living longer (the demographic transition) and their diet and lifestyles are changing (the epidemiological transition), leading to an increase in NCEDs.

The **demographic transition** (the fact that more people are living longer) is taking place due to better nutrition, a safer and healthier environment, improved hygiene and improved health services. Because DR, glaucoma and AMD mainly affect people over the age of 50, the demographic transition has resulted in an increase in the number of people affected by these conditions.

**ABOUT THIS ISSUE**

**Figure:**
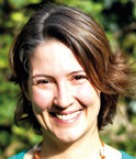
Elmien Wolvaardt Ellison

Editor: *Community Eye Health Journal*.

There have been many successes in the fight against avoidable blindness, and it is encouraging to note the reduction in the number of people visually impaired or blind due to infectious diseases. With these successes, however, come new challenges – one of which is the increase in non-communicable diseases at the back of the eye. Although these diseases are certainly complex and difficult to deal with clinically, the challenges we face are about more than that – they involve both the patient (in terms of their acceptance of, and compliance with, treatment) and the eye health system: that is, everyone and everything involved in eye health delivery. In this issue we will be looking at the clinical challenges, new developments such as anti-VEGF drugs, and the health system. Our next issue (Issue 88) will address patient behaviour.

High-income countries have already experienced this transition. Middle-income countries are rapidly undergoing this change, whereas it is just starting to happen in low-income countries.

The **epidemiological transition** means a change in population health due to changes in lifestyle. Non-communicable diseases, particularly cardiovascular disease and diabetes (with its complications, including diabetic retinopathy) are increasing because of changes in how people live. People are less active, eat more and eat less healthily – mainly as a result of urbanisation. Smoking, a risk factor for AMD, remains prevalent in many populations.

As with the demographic transition, the epidemiological transition has already occurred in high-income countries. Middle-income countries (mainly in Asia and Latin America) are currently undergoing this transition, and it is already starting to become noticeable in low-income countries.

## Will we be able to cope with these diseases?

In many high-income countries, progress has been made in the provision of services for the prevention, early detection and management of DR, glaucoma and AMD. The countries that have been successful at dealing with these diseases have done so by providing comprehensive eye care services that are integrated into national health systems.

‘Mainly due to urbanisation, people are less active, eat more and eat less healthily’

In addition, to enhance the quality of life and promote the independence and inclusion of people with permanent, severe vision loss, they have prioritised the provision of low vision services and rehabilitation. Most low-income countries, and some middle-income countries, however, are not able to provide adequate services to prevent and manage these diseases. There is a shortage of eye care professionals and many of these do not have the level of skill and resources needed to effectively handle these eye conditions. In addition, many eye health services in these countries lack an interdisciplinary, patient-centred approach, which is critical for the successful management of NCEDs.

## What is the way forward?

The WHO Global Action Plan is structured along three objectives, all of which relate to NCEDs in the following ways:

1. **Advocacy based on evidence.** There is an urgent need for advocacy about NCEDs at both global and local levels (in other words, arguing on behalf of NCEDs so that these diseases, and the people affected by them, get the attention they deserve).
  **Figure:**
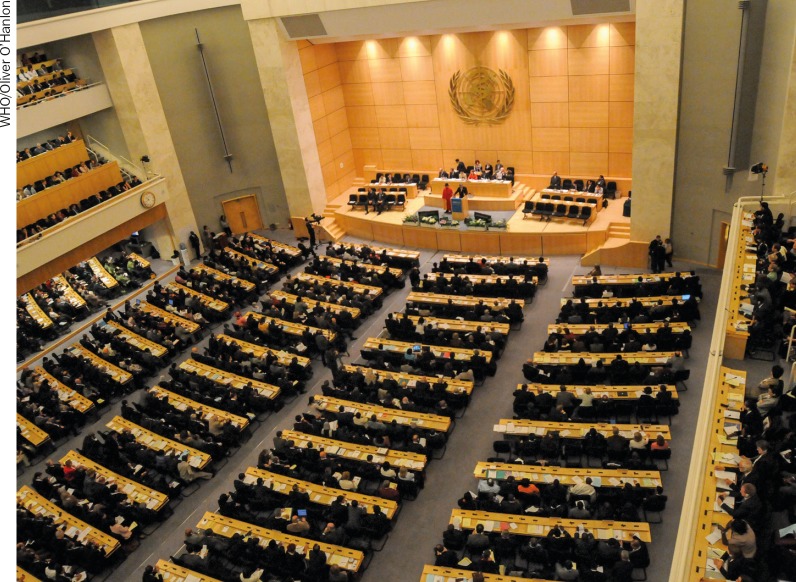
The World Health Assembly opens in Geneva
2. **Policy.** There are areas that need to be addressed at policy level, for example: universal access through health insurance; development of sufficient and adequately trained human resources for eye health; provision of comprehensive eye care; sustainable financing of eye care; provision of essential eye medicines; and monitoring of the key eye health indicators.
3. **Multi-sectoral engagement.** This refers to the engagement between the health sector and non-health sectors. Multi-sectoral engagement, for example with education, environment, agriculture, transport, trade etc. is particularly important for reducing the risks of diabetes, and therefore the risk of DR.

## What are the priorities for action?

We now need to prepare to deal with NCEDs. Our chances of success depend on the availability of committed, sufficiently trained eye care professionals who have the equipment they need and are working within an adequate infrastructure. This means that eye health professionals must be trained to provide comprehensive eye examinations and be confident in managing NCEDs, including the provision of patient counselling.

In order to use the available eye care professionals efficiently, it is necessary that they work in teams. The composition of the team and the distribution of tasks among team members depend on local and/or national regulations.

The necessary equipment and infrastructure (including good record-keeping processes) need to be put in place so that, once trained, eye care providers can examine patients, manage them, and follow them up.

With NCEDs, the need for appropriate and often life-long follow-up means that eye care providers must be able to make informed clinical decisions. These decisions will have a major long-term impact on patients, both in terms of their time and compliance with the treatment regime, and in terms of the lifelong costs of managing their condition. Establishing health insurance-based financing of health care – which includes eye care – appears to be the optimal way to prevent vision loss in those individuals who may not be able to afford out-of-pocket payments.

Patients must be able to comply with the treatment regimes, which means that the medicines they need have to be available and affordable. This means that the procurement and distribution logistics of eye medicines may require review. One can learn from approaches implemented by large intervention programmes for HIV/AIDS, TB and malaria, using bulk purchasing to drive prices down and to ensure quality control and standardisation.

Many individuals affected by NCEDs will need low vision services at some point during their lives, but the availability and affordability of these services have been neglected. Even in high-income countries, there may be uneven coverage of the population: most low vision services are provided in urban areas, resulting in limited access for some. Adequate low vision and rehabilitation services should therefore be a significant part of comprehensive eye care. Once people have lost their sight due to an NCED, there is usually no way to restore vision, so rehabilitation and low vision are currently the only remaining intervention.

*Universal eye health: a global action plan 2014–2019*, as endorsed by the World Health Assembly in 2013, now serves as a road map for the development of comprehensive eye care services integrated into national health systems. Attainment of universal access to eye health will not be possible without adequate attention to the development of eye care services addressing NCEDs.

**WHO Global Action Plan**

“*Substantial reduction of avoidable visual impairment depends on progress in other global health and development agendas, such as the development of comprehensive health systems, human resources for health development, improvements in the area of maternal, child and reproductive health, and the provision of safe drinking water and basic sanitation. Eye health should be included in broader non-communicable and communicable disease frameworks, as well as those addressing ageing populations. The proven risk factors for some causes of blindness (e.g. diabetes mellitus, smoking, premature birth, rubella and vitamin A deficiency) need to be continuously addressed through multi-sectoral interventions*.”

– *Universal eye health: a global action plan 2014-2019*
